# Phenotypic and Genotypic Correlation of Antimicrobial Susceptibility of Bacteroides fragilis: Lessons Learnt

**DOI:** 10.7759/cureus.36268

**Published:** 2023-03-16

**Authors:** Lisha Jha, Binesh Lal Y, Naveen Kumar D Ragupathi, Balaji Veeraraghavan, John Antony J Prakash

**Affiliations:** 1 Department of Clinical Microbiology, Christian Medical College, Vellore, IND

**Keywords:** b.fragilis, cfia, ermf, nim, genotypic markers, redundant antibiotics, meropenem, metronidazole, antimicrobial resistance

## Abstract

Background

*Bacteroides fragilis* is an opportunistic pathogen causing severe infections, including bacteremia. There have been increased reports of antimicrobial resistance in *B. fragilis*. However, phenotypic testing of susceptibility is time consuming and not cost effective for anaerobes. The present study investigates the correlation of phenotypic susceptibility with genotypic markers; to determine if these could be considered for deciding empirical therapy for *B. fragilis*.

Material and methods

*Bacteroides fragilis* isolates from various clinical samples including exudates, tissue, and body fluids were collected between November 2018 and January 2020 in the Department of Clinical Microbiology, Christian Medical College (CMC) Vellore. Species identification was done by Matrix Assisted Laser Desorption Ionization time of flight mass spectrometry (MALDI TOF) according to the manufacturer’s instructions. A total number of 51 *B. fragilis* isolates were tested against metronidazole, clindamycin, piperacillin/tazobactam, and meropenem phenotypically by agar dilution method using Clinical & Laboratory Standards Institute (CLSI) 2019 guidelines and minimum inhibitory concentrations (MIC) were interpretated. The genotypic markers for antimicrobial resistance genes (*nim*, *emrF,* and *cfiA*) were studied by polymerase chain reaction (PCR) assay as per the standard protocol on all isolates to detect resistance genes.

Results

*B. fragilis* isolates in this study expressed 45%, 41%, and 16% phenotypic resistance to clindamycin, metronidazole, and meropenem, respectively, with least resistance to piperacillin/tazobactam (6%). Among the metronidazole resistant isolates, 52% harbored *nim* gene. *Nim* gene was also present in 76% (23/30) of the metronidazole susceptible isolates. Similarly, *cfiA* was present in all eight meropenem resistant isolates in addition to 22% (9/41) of the susceptible isolates. All *cfiA* negative isolates were phenotypically susceptible. Interestingly, 74% (17/23) of the clindamycin resistant isolates were positive for* ermF.*

Conclusions

Detection of a limited set of genes does not always correlate with phenotypic resistance to metronidazole and clindamycin due to the reported influence of insertion sequence (IS) elements, efflux, and other genetic determinants. Certainly, the absence of the* cfiA *gene can be employed to rule out meropenem resistance. However, redundant use of antibiotics such as meropenem along with metronidazole could be avoided for *B. fragilis*, which might otherwise elevate meropenem resistance. Recommendation of metronidazole requires prior phenotypic testing due to the reported 41% resistance.

## Introduction

Anaerobic bacteria are components of the human intestinal flora that also colonize the oral cavity, upper respiratory tracts, and the female genital tract [1]. *Bacteroides fragilis*, the most virulent and most common Bacteroides species, is an obligate gram negative, non-spore-forming anaerobic bacterium associated with intestinal and extra intestinal infections in humans [2]. Routine diagnosis of anaerobic organisms remains cumbersome and time consuming, requiring a special anaerobic culture environment; also, species identification is challenging as anaerobic organisms are inert to most biochemical tests. But correct identification is important as AST (antibiotic susceptibility testing) varies between organisms [3].

The approach to the treatment of anaerobic infections is mostly empirical, as routine testing is time-consuming and is not cost-effective [4]. The most frequently used antibiotics include metronidazole, β-lactams - beta lactamase inhibitor (BL-BLI) combinations, carbapenems, and clindamycin [5]. However, the *Bacteroides fragilis* group tends to be resistant to these antibiotics. An increasing resistance trend has been reported worldwide in the past two decades [6]. This is the fact that the prevalence of antimicrobial resistance varies both between different geographical areas and also among the different medical centers within the same country [7]. Failure to direct appropriate therapy against anaerobic organisms often leads to clinical failure; therefore, susceptibility testing is necessary to provide data for appropriate empirical antimicrobial therapy and to establish national and regional guidelines [8].

The aim of the study is to determine the minimum-inhibitory concentrations (MIC) for metronidazole, clindamycin, meropenem, and piperacillin-tazobactam for *B. fragilis* and to compare these with genetic markers for antimicrobial resistance: *nim* (metronidazole), *ermF* (clindamycin) and *cfiA* (meropenem). This article was previously posted to the Research Square preprint server on February 15, 2022.

## Materials and methods

Sample collection

*Bacteroides fragilis* was isolated from various clinical samples, including exudates, tissue, and body fluids. Isolates collected between November 2018 and January 2020 in the Department of Clinical Microbiology, Christian Medical College (CMC) Vellore, were included in this study. The Institutional Review Board of Christian Medical College, Vellore, approved "Study on antimicrobial susceptibility pattern in clinical isolates of Bacteroides fragilis " with an approval number 11462. Samples were cultured in anaerobic blood agar and neomycin blood agar and incubated at 37 ˚C for 48-72 hours in anaerobic conditions using the Anoxomat Mark system (Mart Microbiology BV, Lichtenvoorde, Netherlands). Species identification was done by Matrix Assisted Laser Desorption Ionization time of flight mass spectrometry (MALDI TOF) (Vitek MS-DS; bioMérieux, Etoile, France) according to the manufacturer’s instructions.

Antimicrobial susceptibility testing

The minimum inhibitory concentration (MIC) to metronidazole, clindamycin, meropenem, and piperacillin-tazobactam was determined by agar dilution method according to Clinical & Laboratory Standards Institute (CLSI) 2019 guidelines. Brucella agar supplemented with hemin, Vit K1, and 5% v/v laked sheep blood (Sigma -Aldrich, Darmstadt, Germany) was inoculated with 10^5^ CFU/ml per spot of *Bacteroides fragilis* strain (0.5 McFarland). Serial two-fold dilutions of antibiotics were incorporated into media. ATCC 25285 *Bacteroides fragilis* was used as the control strain. Plates were incubated at 37 °C for 48 hours in anaerobic conditions using the Anoxomat Mark system. The lowest concentration of antibiotics that inhibit bacterial growth was considered as the MIC.

Sample storage

After isolating *B. fragilis* isolates from various clinical samples, antimicrobial susceptibility testing was performed. Genomic DNA was extracted from 48-hour pure growth on anaerobic blood agar culture plates. The extracted DNA was stored at -80°C in cryovials until polymerase chain reaction (PCR ) was performed. The maximum duration between extraction and performing PCR was three months. 

Identification of genetic markers for antimicrobial resistance

Genomic DNA was extracted using QIAamp DNA mini kit (QIAGEN GmbH, Hilden, Germany) from 48 hours of pure growth on anaerobic blood agar culture plate. Extraction was done as per the manufacturer’s instruction. 

The 458 bp region of *nim *gene, 466 bp of *ermF* gene, 353 bp of *cfiA *gene were amplified in a VeritiTM Thermal Cycler (Applied Biosystem, Foster City, CA, USA) as described by Boente et al. [1] and Nakano et al. [8] using the following primer sequence: nimF (5ˈ-ATGTTCAGAGAAATGCGGCGTAAGCG-3ˈ); and nimR (5ˈ- GCTTCCTTGCCTGTCATGTGCTC-3ˈ), for clindamycin: ermF F (5ˈ -CGGGTCAGCACTTTACTATTG-3ˈ); and ermF R (5ˈ -GGACCTACCTCATAGACAAG-3ˈ), for meropenem: cfiA F (5ˈ -ATGGTACCTTCCAACGGG-3ˈ); and cfiA R(5ˈ -CACGATATTGTCGGTCGC-3').

Thermal profiles used for *nim* gene were 94 °C for 60 sec, 55 °C for 60 sec, 72 °C for 30 sec for 30 cycles; for ermF gene, 94 °C for 30 sec, 50 °C for 30 sec, 72 °C for 2 min for 35 cycles; for cfiA gene 94 °C for 60 sec, 56 °C for 60 sec, 72 °C for 60 sec for 35 cycles with a final extension of 72 °C for 7 min as described by Nakano et al. [8]. The PCR products were visualized in a 2% agarose gel electrophoresis with a 100 bp ladder.

## Results

A total of 51 non-duplicate *B. fragilis* isolates were obtained during the study period. Most of these isolates (74.5%) were recovered from exudates followed by body fluids. In this study piperacillin/tazobactam had the least resistance (6%), followed by meropenem (15%), metronidazole (41%), and clindamycin (45%). Table 1 lists the interpretation criteria and the observed MICs for the tested antimicrobials against *B. fragilis* isolates.

**Table 1:** MIC distribution of metronidazole, clindamycin, meropenem, and piperacillin/tazobactam for B. fragilis isolates. S, susceptible; I, intermediate; R, resistance; MIC, minimum-inhibitory concentration; n, number of isolates; µg/mL, microgram per millilitre

Drugs	Interpretation			MIC range tested for (µg/mL)	MIC distribution (n = 51)		
	S	I	R		S	I	R
Metronidazole	≤ 8	16	≥32	0.25-64	30	0	21
Clindamycin	≤ 2	4	≥8	0.125-16	25	3	23
Meropenem	≤ 4	8	≥16	0.031-16	41	2	8
Piperacillin/ tazobactam	≤ 16/4	32/4-64/4	≥128/4	0.062-256	46	3	2

*Nim* gene and metronidazole

**Figure 1:**
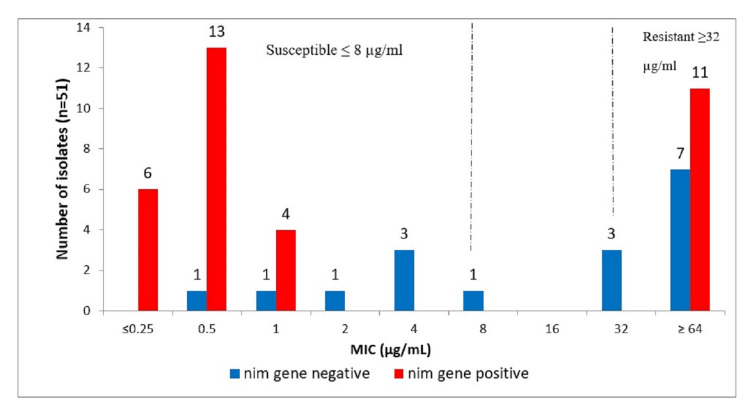
Representation of phenotypic MICs and the gene present for metronidazole resistance (nim gene). MIC, minimum-inhibitory concentration; n, number of isolates; µg/mL, microgram per milliliter

Out of total 51 *B. fragilis* isolates, 21(41%) were resistant (≥32 µg/mL) to metronidazole and 11 (52%) of which contained the *nim* gene. Interestingly, 23 susceptible isolates were also harboring *nim* gene (Figure 1).

*ErmF* gene and clindamycin

**Figure 2:**
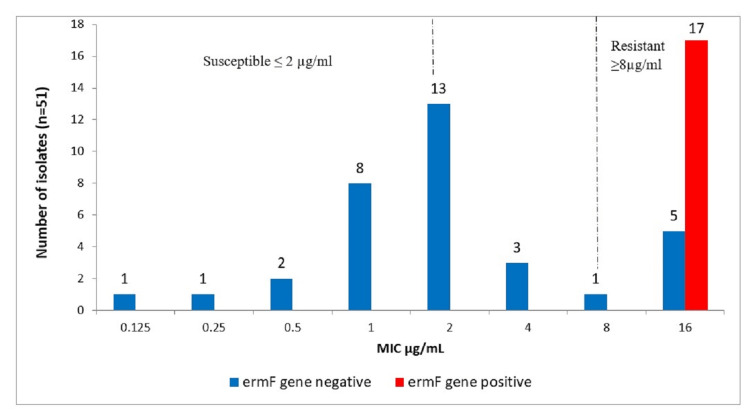
Representation of phenotypic MICs and the gene present for clindamycin resistance (ermF gene). MIC, minimum-inhibitory concentration; n, number of isolates; µg/mL, microgram per millilitre

In this study, 17 (74%) out of 23 resistant isolates (16 µg/ml) were positive for the *ermF* gene. None were present among the susceptible isolates (Figure 2).

*CfiA *gene and resistance to meropenem

**Figure 3:**
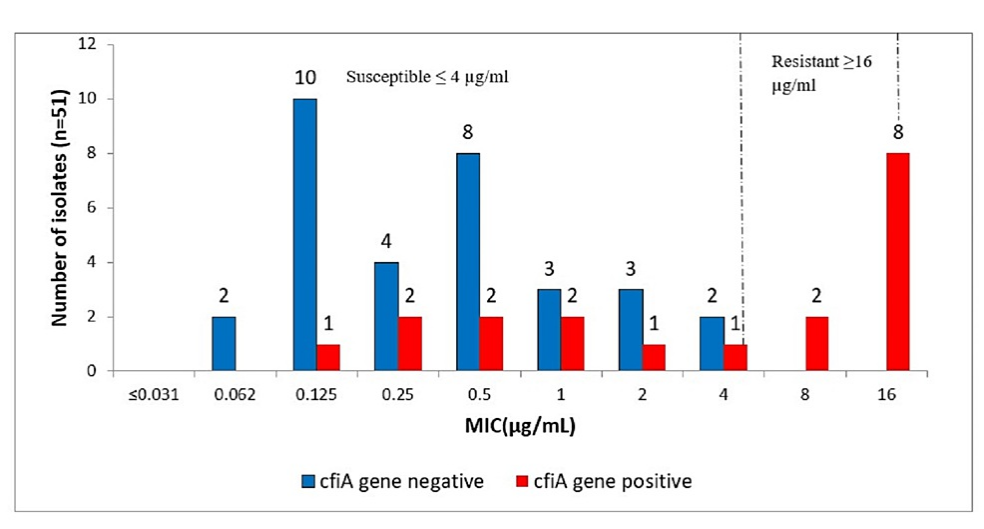
Representation of phenotypic MICs and the gene present for meropenem resistance (cfiA gene). MIC, minimum-inhibitory concentration; n, number of isolates; µg/mL, microgram per millilitre

The *cfiA* gene was present in all eight meropenem resistant isolates. The two intermediate isolates harbored the *cfiA* gene. Remarkably, 22% (9/41) of the susceptible isolates also carried the *cfiA *gene. All *cfiA* negative isolates were phenotypically susceptible (Figure 3).

## Discussion

*Bacteroides fragilis* has been reported for being the cause of a wide range of infections and is often associated with drug resistance. Most patients receive empiric treatment as anaerobic susceptibility test results are unavailable or delayed [9]. Determining MICs by the recommended agar dilution method gives information regarding resistance trends over time [10]. Due to recent developments in molecular microbiology, there are reported PCR methods to identify resistance by genetic determinants rapidly. Though phenotypic testing is more reliable, phenotypic resistance in anaerobes may not always positively correlate with the genotypic findings. This is mainly due to the multiple resistant markers and the varying mechanisms of resistance, including the involvement of insertion sequence (IS) elements for gene regulation [11,12].This study is the first of its kind in India to correlate phenotypic and genotypic resistance mechanisms in *B. fragilis* in order to define the most effective way to treat *B. fragilis *infections. Antimicrobial resistance in* B. fragilis* is mediated by various mechanisms including the production of drug modifying enzymes, efflux of drugs, or inactivation of drugs through the expression of resistance genes [13]. This study utilised* nim* (metronidazole), *ermF* (clindamycin) and *cfiA* (meropenem) genes as molecular markers.

Phenotypic results revealed that 41% of *B. fragilis* were resistant to metronidazole, the commonest drug used for treating anaerobic infections [14]. This is comparable with an Indian study by Sethi et al. [15], which also reported 41% resistance. Another study by Sood et al. from North India reported 57.7% resistance to *B. fragilis *[16].The increased prevalence of metronidazole resistance (41%) reported in this study could be region specific, as antimicrobial resistance varies among regions. In this study, 52% of the metronidazole resistant isolates carried the *nim* gene. This finding is consistent with studies by Vishwanath et al. and Gal and Brazier, which reported positive rates for both phenotypic and genotypic resistance of 50% and 48%, respectively [17,18]. In contrast, Akhi et al. [19] found no prevalence of the nim gene in resistant isolates. This might be due to other mechanisms such as overexpression of the multidrug efflux pump, overexpression of recA or deficiency of feoAB [19]. Interestingly, in a study from Gal and Brazier (2004), 14% of susceptible isolates were reported to carry the *nim* gene. Silent *nim* gene might be due to the absence of an insertion sequence (IS) element promoter region [18]. There is strong evidence that these IS elements carry regulatory signals for the expression of *nim* genes [20]. Studies have shown that silent *nim* genes can be expressed when isolates carrying them are exposed to metronidazole for longer periods of time [21].

The present study reports 45% phenotypic resistance to clindamycin. This high percentage resistance to clindamycin may be due to its widespread use in the treatment of anaerobic infections in intra-abdominal, pelvic, lower respiratory, bone, and skin and soft tissue infections [22].The study data is comparable to the observation by Vishwanath et al. [17], which reported resistance rate of 38% and Sood et al. [16], which reported 46.8% resistance. Further, 74% phenotypically resistant isolates were positive for *ermF*. The *ermF* gene was present only in phenotypically resistant isolates. Similar findings were reported by Kouhsari et al. [23] and Eitel et al. [24], which showed prevalence rates of 76% (206/364) and 74% (23/31) for *ermF* in resistant isolates. It has been observed that the* ermF* gene is frequently present in conjugative transposons. Resistance in these isolates might be due to the other mechanisms conferred by linA, other erm genes such as *ermG/ermS, msrA* or efflux pump mechanism [11].

Meropenem has been reported to have good coverage against anaerobes [25]. In this study, 15% of *B. fragilis* were resistant to meropenem. Similar results have been reported by Jamal et al. and Wang et al., where they found 17% and 19% resistance, respectively [26,27], while Wybo et al. reported slightly reduced resistance rates [28] at 10%. Contrary to this, Vishwanath et al. [17] reported nearly complete absence of resistance for meropenem. Overall, the prevalence of the *cfiA* gene in this study was 37%. All isolates (n = 8) with high MICs (≥ 16 µg/ml) for meropenem were carrying resistance gene. Similar result was reported by Gao et al. in China [29] and Wybo et al. [28]. However, 22% of *cfiA* positive isolates displayed lower levels of meropenem MICs. This might be due to the proven fact that *cfiA* without an upstream insertion sequence (IS) will display lower MICs as opposed to high MICs for *cfiA*s with an upstream IS, which provides a strong promoter for *cfiA *expression [12,29]. All *cfiA* negative isolates were phenotypically susceptible. Based on this observation, it could be interpreted that *cfiA* negative isolates will mostly be phenotypically susceptible. In most cases, meropenem is used for treating *B. fragilis* along with metronidazole. Though both the antimicrobials have good coverage for anaerobes, use of redundant antimicrobials will further add to the existing resistance and complicate antimicrobial stewardship.

β-lactams-beta lactamase inhibitor combinations (BL-BLI) are frequently used for mixed infections (aerobic-anaerobic) as they have a wide range of activity against the majority of anaerobic bacteria [4]. Interestingly, studies from Scandinavian countries and one report from India have reported increased resistance to piperacillin/tazobactam compared to carbapenem [16]. Contrary to these reports, the present study showed *B. fragilis* was highly susceptible to piperacillin/tazobactam as only two out of 51 isolates had high MICs. Our result was concordant with other studies where resistance reported for piperacillin/tazobactam varied from 0-5% [6,17].

Observations from this study and the corresponding literature help to decide empirical therapy for* B. fragilis*. Various local sites can generate antimicrobial resistance (AMR) surveillance data which would help recommend appropriate anaerobic cover for empiric therapy and would avoid the use of redundant antibiotics, thereby saving “watch” antibiotics such as meropenem.

Limitation of our study is that clinical data was not assessed and results were not available in real time for clinical use. Other mechanisms were not explored to understand the cause of discordance between phenotypic and genotypic resistance.

## Conclusions

Anaerobic resistance levels worldwide are generally not on the higher side. For β-lactams - beta lactamase inhibitor combinations, there is observed discrepancy. In some cases, the susceptibility of piperacillin/tazobactam may be increased compared to meropenem, and in other cases, the susceptibility of piperacillin/tazobactam may be decreased compared to meropenem. This study reports reduced carbapenem susceptibility for *B. fragilis* over piperacillin/tazobactam. Unless all resistance mechanisms are analyzed, in addition to the selected genes, evaluating genotypically may not reveal the true correlation with phenotypic results. We have learnt that phenotypic results are more reliable and applicable for the clinical scenario, at least for* B. fragilis*. With varying susceptibility profiles for major drugs, it is imperative to do antimicrobial resistance (AMR) surveillance and to report and recommend the appropriate antimicrobial choice periodically. To facilitate this, improved, less cumbersome, quicker, more economical diagnostic tests need to be developed. Moreover, this study highlights that metronidazole requires antimicrobial susceptibility testing before it can be recommended, as the reported resistance of over 40% is deemed unacceptable for empirical choice.
